# Agronomic performance, oil quality, and multivariate characterization of Anatolian safflower (*Carthamus tinctorius* L.) germplasm under Mediterranean winter-growing conditions

**DOI:** 10.1186/s12870-026-09499-2

**Published:** 2026-07-25

**Authors:** Celal Sayilir, Emre Ilker

**Affiliations:** 1 Department of Field Crops, Graduate School of Natural and Applied Sciences, Ege University, İzmir, Türkiye; 2 Department of Field Crops, Faculty of Agriculture, Ege University, İzmir, 35100 Türkiye

**Keywords:** *Carthamus tinctorius*, Anatolian germplasm, Oil quality, Principal component analysis, Seed yield, Mediterranean environment

## Abstract

Anatolian safflower germplasm represents a potentially promising germplasm for improving seed yield and oil quality under Mediterranean environments; however, information regarding its agronomic performance and fatty acid composition remains limited. This study evaluated the agronomic traits, oil content, fatty acid composition, and multivariate relationships of 16 Anatolian safflower genotypes and five registered cultivars under Mediterranean winter-growing conditions during two consecutive growing seasons. Significant variation was observed among genotypes for most agronomic and quality traits. Among the Anatolian accessions, TR 42670 exhibited superior seed and oil yield performance, TR 50118 showed the highest oil content, and TR 49117 was characterized by high linoleic acid content. The cultivars Asol and Olas were distinguished by their relatively high oleic acid concentrations. These results demonstrated the existence of considerable diversity for both productivity and oil quality traits within the evaluated germplasm. Principal component analysis revealed that fatty acid composition, particularly the oleic–linoleic acid balance, together with yield-related traits, represented the major sources of variation among genotypes. Multivariate analyses clearly separated high-oleic and high-linoleic materials and highlighted substantial diversity among the evaluated accessions.

Overall, the results indicate that Anatolian safflower germplasm contains valuable genetic variation for agronomic performance, oil content, and fatty acid composition. These genetic resources may provide useful parental material for breeding programs aimed at improving productivity and oil quality in Mediterranean environments.

**Supplementary Information:**

The online version contains supplementary material available at https://doi.org/10.1186/s12870-026-09499-2.

## Introduction

Climate change, increasing water scarcity, loss of agricultural land, and continued population growth are placing substantial pressure on agricultural production systems worldwide. These challenges have intensified the need for resilient crop species capable of maintaining productivity under adverse environmental conditions. In this context, alternative oilseed crops have gained increasing attention because of the growing demand for vegetable oils and the vulnerability of conventional cropping systems to climatic stresses [20].

Safflower (*Carthamus tinctorius* L.) is a drought- and salinity-tolerant oilseed crop that performs well under arid and semi-arid conditions [10, 12, 22]. In addition to its adaptability, safflower oil is valued for its high content of unsaturated fatty acids and bioactive compounds with nutritional and industrial importance [13]. Nevertheless, low seed yield and a long growth period remain major constraints limiting the wider adoption of safflower in many production regions [3, 15].

Although numerous studies have evaluated the agronomic performance and oil quality characteristics of commercial safflower cultivars, information regarding the adaptation potential and fatty acid composition of Anatolian safflower germplasm remains limited. Anatolia represents an important source of safflower diversity and possesses promising genetic resources that may contribute to breeding programs targeting yield improvement, oil quality enhancement, and adaptation to environmental stresses [6].

Despite increasing interest in safflower cultivation under Mediterranean environments, comprehensive evaluations of Anatolian gene bank materials under Mediterranean winter-growing conditions remain scarce. Therefore, the objectives of this study were (i) to evaluate the agronomic performance of Anatolian safflower genotypes and registered cultivars under Mediterranean winter-growing conditions, (ii) to determine variation in seed oil content and fatty acid composition among genotypes, and (iii) to identify promising genetic materials that may be utilized in future safflower breeding programs targeting both productivity and oil quality.

## Materials and methods

The sixteen safflower genotypes were selected from the AARI Gene Bank to represent the geographical diversity of Anatolian safflower germplasm, covering different ecological regions, altitudes, and production environments of Türkiye. Five registered cultivars commonly used in safflower breeding and production were included as reference materials for comparison (Tables 1 and 2).

**Table 1:** Safflower genotypes of Anatolian origin used as material in the study

Genotype	Origin (Province)	District	Altitude (m)
TR 42942	Balikesir	Balya	300
TR 51504	Bolu	Goynuk	540
TR 50118	Bursa	Harmancik	780
TR 50120	Bursa	Harmancik	780
TR 49116	Isparta	Gelendost	850
TR 49117	Isparta	Gelendost	850
TR 49118	Isparta	Gelendost	860
TR 42630	Edirne	Havsa	40
TR 42631	Edirne	Havsa	40
TR 42642	Kirklareli	Imampazari	40
TR 42646	Edirne	Uzunkopru	40
TR 42660	Kirklareli	Vize	90
TR 42666	Kirklareli	Vize	160
TR 42667	Kirklareli	Vize	160
TR 42670	Tekirdag	Saray	240
TR 73821	Eskisehir	Gunyuzu	991

**Table 2:** Safflower varieties used as material in the study

Variety	Registrant Institution/Organization
Asol	Sanliurfa Southeastern Anatolia Agricultural Research Institute
Balci	Eskisehir Anatolian Agricultural Research Institute
Olas	Edirne Trakya Agricultural Research Institute
Linas	Edirne Trakya Agricultural Research Institute
Safir	Edirne Trakya Agricultural Research Institute

The field experiment was carried out in the experimental fields of İzmir Ege University Faculty of Agriculture Menemen Research and Application Farm (38°34’45.4 “N, 27°01’23.4 “E) in the 2019–2020 and 2020–2021 growing seasons under field conditions with 4 replications according to the Randomized Complete Block Design.

In the study area, where the typical Mediterranean climate prevails, the first growing season was characterized by higher-than-average precipitation, whereas the second season was drier, particularly during the reproductive period. Total annual precipitation was 518.6 mm in 2020 and 406.4 mm in 2021, compared with the long-term average of 546.7 mm (Table 3).

**Table 3:** Experimental period and long years climate data of the experimental area (AARI 2022)

Months	Climate Data Of The Trial Years (December 2018 to January 2022)									LONG YEARS CLIMATE DATA (1954–2019)		
	Average Temperature (C°)			Total Precipitation (mm)			AverageHumidity (%)			AverageTemperature (C°)	TotalPrecipitation (mm)	Average Humidity (%)
	2019	2020	2021	2019	2020	2021	2019	2020	2021			
January	8.16	7.55	10.50	310.20	47.20	164.00	76.28	64.85	73.31	7.83	97.99	66.95
February	9.28	9.90	10.70	105.00	72.80	62.60	70.42	69.12	66.26	8.95	73.86	64.29
Mart	12.28	12.71	10.36	33.60	62.40	129.60	62.04	67.39	65.19	11.17	62.29	62.43
April	15.14	15.18	15.78	57.60	52.20	33.20	61.32	59.87	63.21	15.12	41.22	59.26
May	20.51	20.74	21.61	3.60	48.60	0.20	59.23	58.79	56.57	20.10	27.27	55.80
June	26.34	24.06	24.87	27.60	34.20	16.80	55.01	60.14	55.14	24.74	9.79	49.65
July	27.02	28.45	29.30	20.60	0.00	0.00	51.35	50.25	47.30	27.06	2.58	47.44
August	28.55	27.79	28.60	0.00	0.00	0.00	47.81	50.28	48.50	26.57	2.51	49.23
September	23.28	25.62	23.60	28.00	0.00	2.80	58.18	57.03	50.65	22.40	12.49	55.11
October	19.69	20.16	17.90	29.40	25.40	62.20	72.17	68.56	63.50	17.51	38.76	61.07
November	16.55	13.70	15.69	59.20	3.00	54.00	74.12	57.67	71.20	13.04	73.89	65.24
December	10.35	12.44	10.80	65.60	172.80	54.00	75.53	74.76	74.19	9.62	104.04	68.12
Mean	18.10	18.19	17.59	61.70	43.22	67.73	63.62	61.56	63.28	17.01	45.56	58.72
Total	217.15	218.32	123.12	740.40	518.60	406.40	763.45	738.71	379.69	204.10	546.70	704.58

The experimental soil had a sandy-loam texture, low salinity, slightly to moderately alkaline pH, low organic matter and phosphorus content, and adequate potassium levels. Detailed soil properties are presented in Table 4. Each experimental plot consisted of five rows, 3 m in length, with 20 cm row spacing and 8 cm intra-row spacing. The length of each block was 21 m and the area was 63 m². A gap of 2.5 m was left between the blocks. In the plots, the 1st and 5th rows were bordered and the plants in the middle three rows (1.8 m²) in each plot were harvested. Sowing was done on November 12, 2019 in the first year and on November 17, 2020 in the second year. Fertilizer rates were determined according to regional recommendations for safflower production and soil fertility status of the experimental area. In both years of the experiment, 30 kg of pure nitrogen, 30 kg of phosphorus and 30 kg of potassium per hectare were applied as base fertilizer before sowing and 42 kg of pure nitrogen per hectare was applied as top fertilizer before the second hoeing. Thinning, hoeing, and disease and pest control were carried out as needed. Harvesting was carried out between 03 and 09 July 2020 in the first year and 05–10 July 2021 in the second year.

**Table 4:** Soil characteristics of the experimental area (Altitude :5 m, Land Type: Valley, Relief: Alluvial Plain)

Feature	Horizon and Depth		
	Ap (0–41 cm)	C1 (41–90 cm)	C2 (90–125 cm)
pH	7.55	7.75	8.24
EC (µS/cm)	308	705	123.3
CaCO_3_ (%)	9.67	6.96	6.19
Sand (%)	64	86	84
Mile (%)	23.28	9.28	9.28
Clay (%)	12.72	4.72	6.72
Composition	Sandy loam	Loamy Sand	Loamy Sand
Organic Matter (%)	1.84	1.53	0.88
% Total Nitrogen	0.101	0.067	0.073
P (ppm)	3.12	2.16	1.57
Ca (ppm)	3160	2772	2772
K (ppm)	155.2	67.9	77.6
Na (ppm)	38.4	28.8	28.8
Mg (ppm)	405.7	270.6	285.2
Fe (ppm)	3.14	2.63	2.09
Cu (ppm)	0.37	0.27	0.19
Zn (ppm)	1.62	0.59	0.24
Mn (ppm)	2.19	1.00	0.76

Observations were made on 10 plants randomly selected from each plot. Plant height (cm), number of lateral branches (number), number of heads (number), number of seeds per head (number), head diameter (cm), thousand seed weight (g), 50% flowering days (days), maturation days (days), seed yield (kg ha^− 1^), harvest index (%), oil ratio (%), oil yield (kg ha^− 1^), fatty acid composition (%) (linoleic, oleic, palmitic and stearic acid ratio) were analyzed. Seed oil content was determined using a SupNIR-2700 Near Infrared Analyzer (Unity Scientific, USA) at the Department of Field Crops, Faculty of Agriculture, Ege University. Oil content was expressed as a percentage of seed dry weight [23, 25].

For fatty acid composition analysis, at least 50 g of seed sample obtained from each plot was submitted to the quality control laboratory of Abalıoğlu Yağ Sanayi A.Ş. Fatty acid composition was determined using an Agilent 7890B gas chromatograph according to the AOCS Ce 1-62-09 method. Fatty acid methyl esters (FAMEs) were analyzed using a Supelco 2380 capillary column (60 m × 0.25 mm × 0.20 μm) equipped with a flame ionization detector (FID). Helium was used as the carrier gas at a linear flow rate of 20 cm s⁻¹. The injector, oven, and detector temperatures were maintained at 250, 185, and 260 °C, respectively. A 1 µL FAME sample was injected with a split ratio of 1:100. Individual fatty acids were identified by comparing their retention times with those of reference standards, and quantified using methyl nonadecanoate as an internal standard [5].

Statistical analyses were performed using TOTEMSTAT (Ver. 2) software [2]. Data from the two growing seasons were subjected to combined analysis of variance (ANOVA) according to a randomized complete block design. In the combined analysis, years and genotypes were considered fixed effects, while blocks were nested within years. The statistical model used was:

$$\mathrm{Yijk}=\upmu+\mathrm{Yi}+\mathrm{Gj}+(\mathrm{YG})\mathrm{ij}+\mathrm{Bk}(\mathrm{Yi})+\varepsilon\mathrm{ijk}$$

where µ is the overall mean, Yi is the effect of year, Gj is the effect of genotype, (YG)ij is the year × genotype interaction, Bk(Yi) is the effect of block within year, and εijk is the experimental error.

Prior to analysis, the assumptions of ANOVA, including normality of residuals and homogeneity of variances, were evaluated. Mean comparisons were performed using the Least Significant Difference (LSD) test at the 0.05 probability level [19].

## Results and discussion

### Yield and yield components

The analysis of variance revealed significant effects of year, genotype, and year × genotype interaction for most yield-related traits (Table 5). The drier conditions during flowering and seed filling in 2021 reduced plant height, head number, thousand-seed weight, seed yield, and maturity duration, indicating differential genotype responses to environmental variation.

**Table 5:** Analysis of variance, mean values and LSD groups for yield and some yield components

Variety / Genotype		Plant Height (cm)		Number of Heads		Number of Seeds Per Head	Thousand Seed Weight (g)		Seed Yield (kg ha^− 1^)		Maturation Days (day)	
		2020	2021	2020	2021	Mean of 2020 and 2021 years	2020	2021	2020	2021	2020	2021
Asol		120.23 bcd	113.45 c-g	11.70 cd	10.70 cd	25.40 i	36.37 bcd	32.69 d	1638.4 ef	1408.4 efg	222.25 a	215.25 a
Balci		125.30 bc	119.57 a-e	10.34 ef	9.93 def	27.79 gh	35.30 cde	33.30 bcd	1287.0 ij	1282.7 ghi	223.25 abc	215.00 a
Linas		135.16 a	125.37 a	12.07 c	11.67 bc	25.04 i	41.45 a	36.76 a	2378.6 a	2326.7 a	223.50 abc	215.50 a
Olas		121.93 bc	119.86 a-e	16.27 a	14.23 a	29.64 c-h	35.77 b-e	35.11 abc	2350.0 ab	2281.6 a	222.25 a	215.25 a
Safir		127.90 ab	115.23 b-g	13.50 b	12.15 b	29.52 d-h	36.81 bc	31.79 de	1927.6 c	1665.8 cd	222.75 ab	217.50 b
TR 42630		125.16 bc	126.23 a	10.52 def	11.77 bc	30.85 cd	27.45 ghi	29.91 e	1093.3 k	1151.9 ij	223.25 abc	222.50 efg
TR 42631		117.83 cde	123.06 ab	8.47 ghi	10.13 def	30.06 cde	28.84 fgh	27.34 fg	1149.7 jk	982.1 k	224.50 c	222.25 efg
TR 42642		121.56 bc	119.46 a-f	8.27 hi	9.33 efgh	29.78 c-g	25.81 i	26.42 g	1132.8 k	1110.0 jk	224.25 bc	222.75 fg
TR 42646		122.76 bc	120.46 a-d	8.04 hij	9.50 d-h	30.44 cde	37.91 b	29.68 ef	1418.8 ghi	1512.5 ef	223.50 abc	217.25 b
TR 42660		127.63 ab	120.20 a-e	9.77 fg	8.93 fghi	31.63 c	35.66 b-e	31.50 de	1770.7 de	1522.3 e	222.50 a	214.00 a
TR 42666		123.13 bc	121.30 abc	6.87 j	7.00 j	28.80 e-h	34.43 de	29.60 ef	1320.4 i	1175.5 ij	222.75 ab	214.25 a
TR 42667		127.13 ab	113.43 c-g	6.84 j	8.53 ghi	24.65 i	36.90 bc	32.96 bcd	1666.8 def	1423.5 ef	222.50 a	214.25 a
TR 42670		126.55 b	114.22 c-g	8.83 ghi	7.63 ij	35.15 b	30.77 f	31.73 de	2214.9 b	1898.2 b	223.25 abc	218.50 bc
TR 42942		125.00 bc	115.86 b-g	7.73 ij	9.63 d-g	35.60 ab	25.32 i	22.88 h	1535.6 fg	1382.1 fgh	222.25 a	221.25 def
TR 49116		121.76 bc	112.50 d-g	9.23 fgh	8.60 ghi	37.22 a	25.30 i	27.08 g	1709.6 de	1383.6 fgh	227.50 de	221.00 de
TR 49117		110.07 e	108.36 g	9.60 fg	9.43 d-h	28.67 e-h	33.54 e	30.29 e	1919.7 c	1854.3 b	228.00 def	220.25 d
TR 49118		120.66 bcd	115.60 b-g	11.23 cde	9.92 def	35.48 ab	25.51 i	29.79 e	1310.4 i	1262.5 hi	229.25 f	222.25 efg
TR 50118		113.00 de	112.16 efg	11.60 cde	9.81 d-g	27.98 fgh	26.36 i	25.71 g	1802.8 cd	1780.5 bc	226.50 d	222.75 fg
TR 50120		125.13 bc	111.40 fg	11.23 cde	9.29 e-h	31.27 cd	29.66 fg	32.79 cd	1393.7 hi	1236.8 ij	229.00 ef	223.00 g
TR 51504		124.52 bc	111.05 g	12.13 c	10.35 de	29.92 c-f	26.67 hi	25.03 gh	1529.5 fgh	1399.9 e-h	222.75 ab	222.75 fg
TR 73821		126.40 b	114.33 c-g	9.56 fg	8.23 hij	27.65 h	30.90 f	35.17 ab	1555.5 fg	1531.9 de	222.50 a	220.00 cd
Mean (varieties)		126.11	120.90	12.78	11.73	27.48	37.14	33.93	1916.3	1793.1	222.80	215.70
Mean (Anatolian genotypes)		122.40	115.54	9.37	9.26	30.95	30.07	29.24	1532.8	1413.0	224.64	219.94
Mean (all genotypes)		123.28	116.82	10.18	9.85	30.12	31.75	30.36	1624.1	1503.5	224.20	218.93
Source of Variation	Year	**		*		**	**		**		**	
	Genotype	**		**		**	**		**		**	
	Year*Genotype	*		**		ns	**		**		**	
	LSD _0.05_	8.148		1.315		2.037	2.360		138.50		1.552	
	Coefficient of Variation (%)	4.86		9.39		6.84	5.44		6.34		0.55	

Means followed by the same letter within each column are not significantly different according to the Least Significant Difference (LSD) test at *P* ≤ 0.05

*ns* non significant

* significant at the *p* ≤ 0.05 level, ** significant at the *p* ≤ 0.01 level

Plant height varied significantly among genotypes and years, ranging from 108.36 cm (TR 49117) to 135.16 cm (Linas) (Table 5). Commercial cultivars were generally taller than Anatolian accessions, although TR 42630, TR 42631, and TR 42666 exhibited comparable plant heights. Values were within the range previously reported for Mediterranean environments [15, 16]. The reduced plant height observed in 2021 suggests that drought stress limited vegetative growth during stem elongation and reproductive development.

The number of heads per plant differed significantly among genotypes and years (Table 5). Olas and Safir consistently produced the highest head numbers, whereas most Anatolian genotypes produced fewer heads. Since head number is a major determinant of safflower yield [21], the superior performance of these cultivars partly explains their high productivity. The reduction in head number under drier conditions indicates the sensitivity of reproductive development to water availability. Nevertheless, TR 42630 and TR 51504 maintained head numbers comparable to those of commercial cultivars, suggesting adaptation to water-limited Mediterranean environments.

The number of seeds per head was not significantly affected by the year × genotype interaction (Table 5), indicating greater genetic control and environmental stability than other yield components. Values ranged from 24.65 seeds head⁻¹ (TR 42667) to 37.22 seeds head⁻¹ (TR 49116), with Anatolian genotypes generally exceeding registered cultivars. TR 49116, TR 42942, TR 49118, and TR 42670 exhibited particularly high seed numbers and may represent useful parental materials for breeding programs [4, 7].

Thousand-seed weight varied significantly among genotypes and years (Table 5). Linas consistently produced the highest seed weight, whereas several Anatolian genotypes showed marked reductions under drought conditions. Compared with seed number per head, thousand-seed weight appeared more environmentally sensitive, suggesting that assimilate availability during grain filling strongly influenced final seed weight. The lower thousand-seed weight of many Anatolian accessions partly explains their reduced seed yield despite producing relatively high numbers of seeds per head.

Seed yield was significantly affected by year, genotype, and year × genotype interaction (Table 5). Seed yield ranged from 1093.3 to 2378.6 kg ha⁻¹ in 2020 and from 982.1 to 2326.7 kg ha⁻¹ in 2021. Linas and Olas consistently produced the highest yields across both seasons. However, several Anatolian accessions, particularly TR 42670, TR 49117, and TR 50118, maintained relatively high productivity under the drier conditions of 2021 and outperformed some registered cultivars. Their stable performance suggests the presence of adaptive traits that help maintain reproductive growth and seed filling under terminal drought stress. Such genotypes may therefore represent promising germplasm for breeding programs targeting Mediterranean environments characterized by irregular rainfall and seasonal drought. The observed yield levels were comparable to those reported for Mediterranean environments [4, 15, 16, 24].

Days to maturity differed significantly among genotypes and years (Table 5). Plants matured approximately one week earlier during the drier second season, resulting in a significant year × genotype interaction. Registered cultivars generally matured earlier than Anatolian accessions. The accelerated maturation observed in 2021 likely reflects a drought-avoidance strategy under moisture-limited conditions. Since prolonged growth duration has been identified as a major constraint to safflower cultivation in Mediterranean environments [3, 15], genotypes combining relatively early maturity with acceptable seed yield may provide useful parental materials for future breeding programs.

### Oil ratio, oil yield and fatty acid composition

Oil content, oil yield, and fatty acid composition were significantly affected by genotype, year, and, for several traits, genotype × year interaction (Table 6), indicating substantial diversity for seed quality traits among the evaluated materials.

**Table 6:** Analysis of variance, mean values and LSD groups for oil ratio, oil yield and fatty acid composition

Variety / Genotype		Oil Ratio		Oil Yield (kg ha^− 1^)		Linoleic Acid Ratio		Oleic Acid Ratio		Palmitic Acid Ratio		Stearic Acid Ratio	
		2020	2021	2020	2021	2020	2021	2020	2021	2020	2021	2020	2021
Asol		33.06 c	30.24 k	541.6 de	426.2 def	38.99 j	41.69 j	51.17 a	48.01 a	5.92 a	6.12 a	1.91 a	2.28 a
Balci		34.46 a	32.48 de	443.6 hi	416.7 ef	64.59 h	64.59 h	25.28 c	25.21 c	6.72 cdef	6.62 bcde	2.29 b	2.30 a
Linas		32.02 d	31.89 ef	761.7 a	742.1 a	74.22 b	76.94 a	14.70 fg	12.72 k	6.65 cde	6.36 abc	2.81 fg	2.91 gh
Olas		33.77 b	31.68 fg	793.6 a	722.9 a	45.31 i	47.58 i	44.86 b	42.25 b	6.19 ab	6.15 a	1.97 a	2.31 a
Safir		33.71 b	33.22 bc	649.6 b	553.3 b	45.63 i	47.15 i	43.99 b	42.24 b	6.16 ab	6.12 a	2.60 b-e	2.67 b-e
TR 42630		29.53 i	31.51 fgh	323.1 k	362.4 gh	74.05 b	74.84 cd	15.90 e	14.89 fgh	6.28 b	6.07 a	2.60 b-e	2.80 efg
TR 42631		31.44 de	32.85 cd	361.3 jk	322.7 h	71.42 f	73.93 def	18.14 d	15.34 fgh	6.25 b	6.39 abc	2.47 bcd	2.74 c-g
TR 42642		30.53 fg	33.28 bc	345.8 k	369.2 g	75.99 a	74.97 cd	13.42 hi	14.31 hij	6.21 ab	6.69 cde	2.74 efg	2.88 fg
TR 42646		30.89 ef	31.42 f-i	438.1 hi	475.4 c	71.71 ef	75.16 c	15.53 ef	13.66 ijk	7.49 fgh	6.43 abc	3.29 k	2.97 h
TR 42660		29.60 hi	30.58 jk	524.0 ef	465.8 cd	71.10 f	70.74 g	18.48 d	18.45 d	6.52 cd	6.45 abc	2.65 c-f	2.79 efg
TR 42666		30.58 fg	31.07 hij	403.1 ij	365.1 gh	73.06 bcd	73.02 f	16.23 e	16.60 e	6.47 cd	6.22 ab	2.69 d-g	2.75 d-g
TR 42667		29.77 hi	30.20 k	496.4 fg	430.1 def	70.89 f	73.36 ef	17.75 d	15.79 ef	6.78 d-g	7.00 e	2.81 fg	2.65 a-d
TR 42670		28.76 j	30.89 ij	636.9 b	586.3 b	71.94 def	74.47 cde	18.20 d	15.43 fg	5.87 a	6.08 a	2.32 bc	2.61 abc
TR 42942		31.08 ef	33.56 ab	477.3 gh	463.7 cd	72.81 cde	74.88 cd	16.36 e	14.42 g-j	6.25 b	6.16 a	2.88 hi	2.73 c-f
TR 49116		30.17 gh	32.29 de	515.8 efg	446.9 cde	69.14 g	73.355 ef	18.92 d	15.32 fgh	7.93 h	6.72 cde	2.99 j	3.05 i
TR 49117		29.92 hi	31.25 ghi	574.5 cd	579.3 b	76.26 a	76.56 ab	13.32 i	12.71 k	6.34 c	6.41 abc	2.74 efg	2.80 efg
TR 49118		30.70 fg	31.58 fgh	402.2 ij	398.5 fg	73.35 bc	73.77 def	14.45 gh	14.84 fgh	6.65 cde	6.27 ab	2.85 gh	2.86 fg
TR 50118		32.83 c	32.81 cd	591.7 c	584.1 b	75.54 a	75.46 bc	14.34ghi	14.93 fgh	6.16 ab	6.14 a	2.67 c-f	2.90 fgh
TR 50120		30.89 ef	31.52 fgh	430.3 i	389.8 fg	71.16 f	73.90 def	16.28 e	14.68 ghi	7.51 fgh	6.48 a-d	3.31 k	2.97 h
TR 51504		31.34 e	33.91 a	479.1 gh	474.8 c	71.69 ef	75.57 bc	15.80 e	13.37 jk	7.56 gh	6.87 de	2.95 ij	2.66 b-e
TR 73821		28.32 j	30.00 k	440.5 hi	459.7cde	71.81 ef	75.56 bc	16.12 e	13.36 jk	7.01efg	6.38 abc	2.85 gh	2.49 ab
Mean (varieties)		33.41	31.91	638.0	572.3	53.75	55.59	36.00	34.08	6.32	6.27	2.31	2.49
Mean (Anatolian genotypes)		30.40	31.80	465.0	448.4	72.52	74.34	16.20	14.88	6.70	6.42	2.80	2.79
Mean (all genotypes)		31.12	31.82	506.2	477.9	68.12	69.88	20.91	19.45	6.61	6.38	2.68	2.72
Source of Variation	Year	**		**		**		**			**		*
	Genotype	**		**		**		**			**		**
	Year*Genotype	**		**		**		**			**		**
	LSD _0.05_	0.604		43.21		1.221		1.072			0.412		0.161
	Coefficient of Variation (%)	1.37		6.28		0.88		2.63			3.14		2.95

Means followed by the same letter within each column are not significantly different according to the Least Significant Difference (LSD) test at *P* ≤ 0.05

* significant at the *p* ≤ 0.05 level, ** significant at the *p* ≤ 0.01 level

Seed oil content ranged from 28.32% to 34.47% (Table 6). TR 50118 exhibited the highest average oil content, followed by Balcı and TR 49117, whereas TR 42631 and TR 42642 showed the lowest values. Several Anatolian accessions equaled or exceeded the oil content of commercial cultivars, highlighting their breeding potential. Oil content values were within the range previously reported under Mediterranean conditions [15], suggesting the presence of favorable alleles for oil accumulation within Anatolian germplasm.

Oil yield, which reflects the combined effects of seed yield and oil content, showed greater variation than oil content alone (Table 6). The highest oil yields were obtained from TR 42670 and Linas. The superior performance of TR 42670 is particularly noteworthy because it combined high seed yield with above-average oil content, emphasizing the importance of simultaneously considering agronomic and quality traits in breeding programs.

Fatty acid composition varied considerably among genotypes and years (Table 6). Linoleic acid was the predominant fatty acid, ranging from 61.30% to 81.58%, whereas oleic acid ranged from 8.59% to 29.76%. Asol and Olas consistently exhibited the highest oleic acid contents, while TR 49117 and TR 50118 were characterized by elevated linoleic acid levels. These results confirm substantial diversity for oil quality traits within the evaluated germplasm.

A strong inverse relationship between oleic and linoleic acids was observed (Table 6), reflecting the biochemical conversion of oleic acid into linoleic acid via the activity of the microsomal oleate desaturase (FAD2). Similar relationships have been widely reported in safflower and are recognized as one of the principal determinants of oil quality variation [1, 11]. The coexistence of high-oleic and high-linoleic genotypes within Anatolian germplasm provides opportunities for developing cultivars targeting different industrial and nutritional uses.

**Fig. 1:**
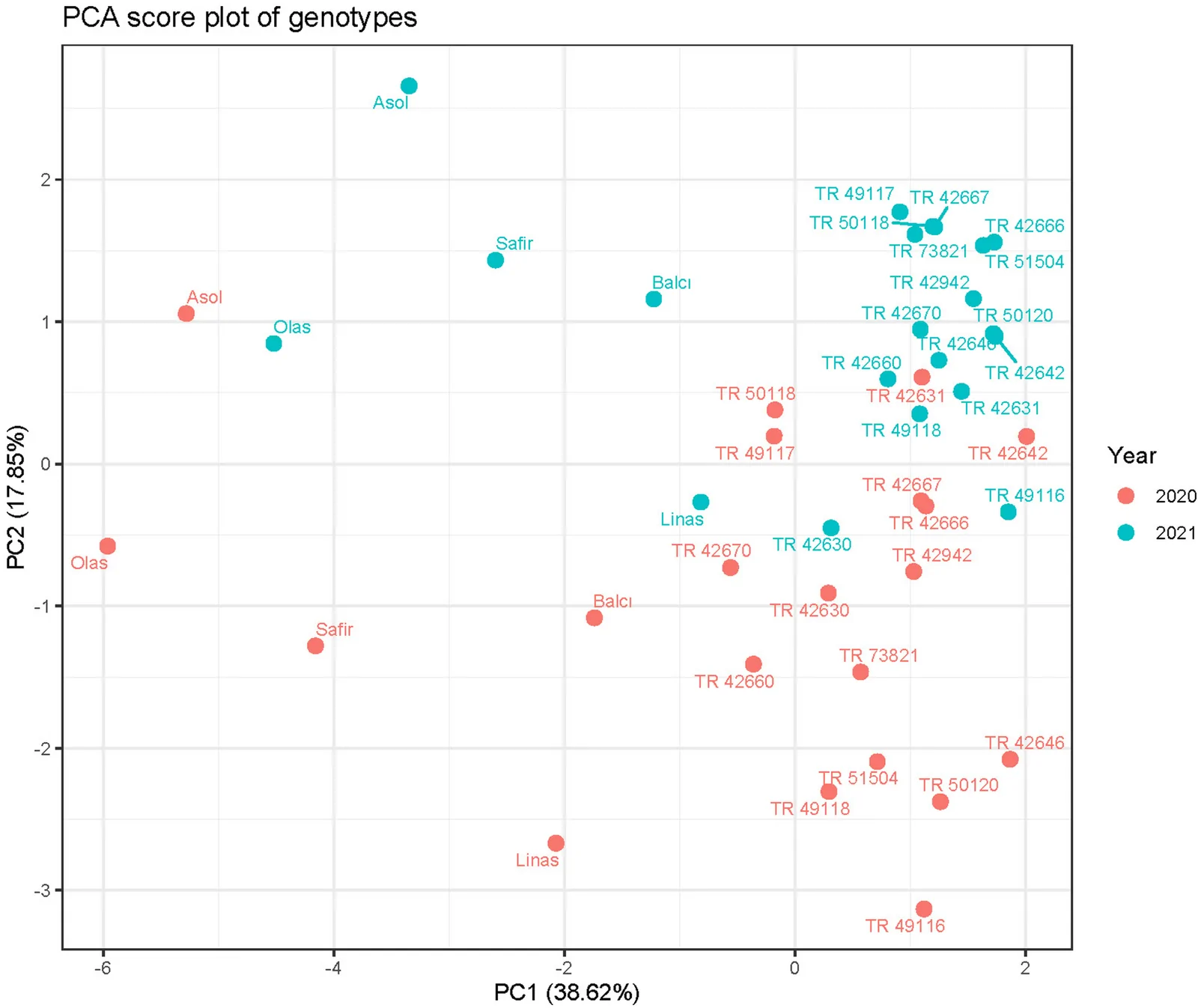
PCA score plot illustrating the distribution of safflower genotypes evaluated in 2020 and 2021 according to the first two principal components

**Fig. 2:**
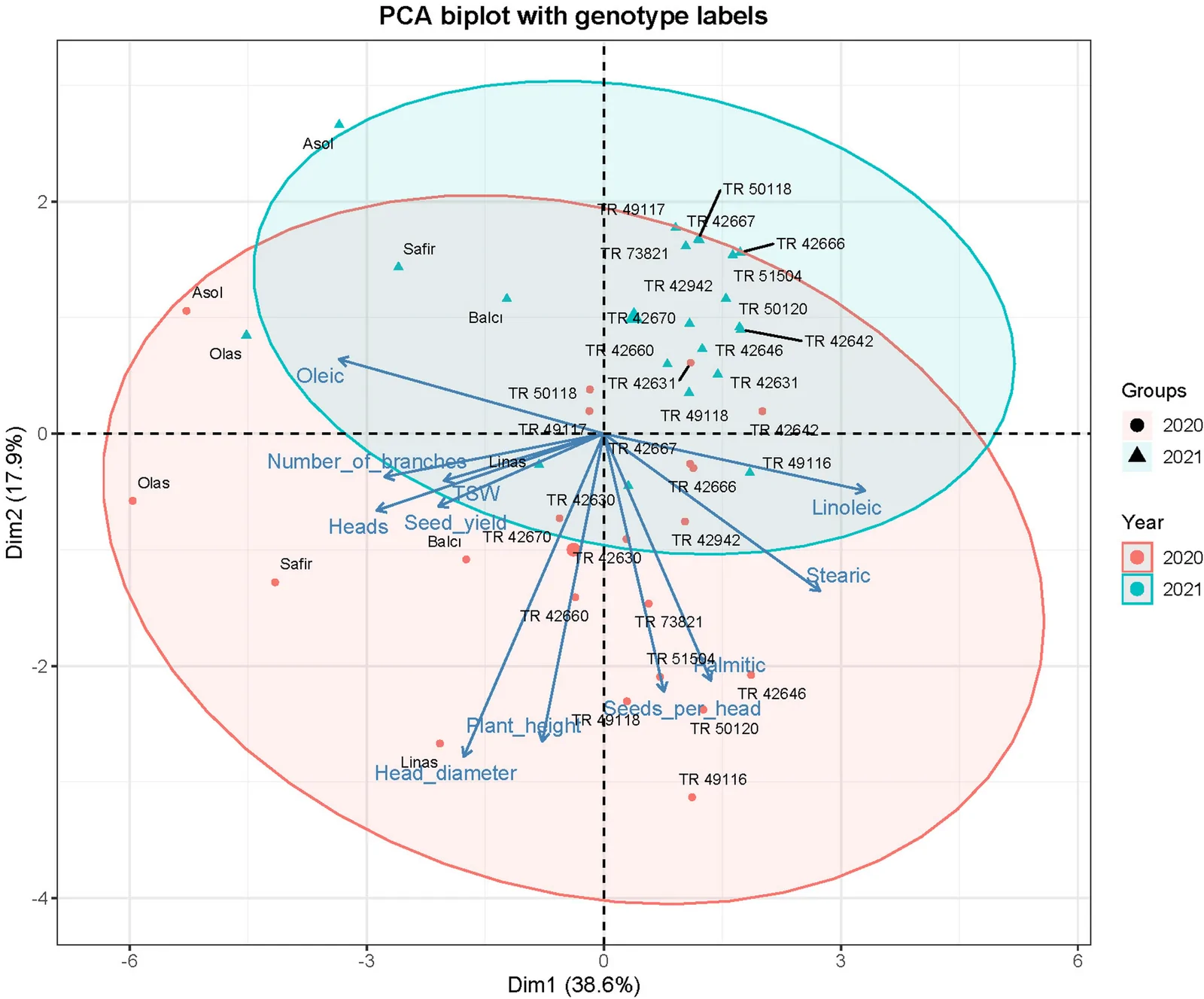
PCA biplot showing the relationships between safflower genotypes and measured agronomic and quality traits across two growing seasons

The importance of fatty acid composition was further confirmed by principal component analysis, where oleic and linoleic acids exhibited opposite loadings on PC1 and clearly separated genotypes in the PCA biplot (Figs. 1 and 2). This result indicates that fatty acid composition was one of the primary sources of variation among the evaluated materials.

Palmitic acid content ranged from 5.57% to 7.49%, whereas stearic acid varied between 1.84% and 3.16% (Table 6). Compared with oleic and linoleic acids, these saturated fatty acids exhibited a narrower range of variation, suggesting greater genetic stability and lower environmental sensitivity, consistent with recent findings in safflower germplasm [14].

Environmental conditions also influenced oil quality traits. Year-to-year differences were particularly evident for oleic and linoleic acid contents (Table 6), indicating that climatic conditions during flowering and seed filling affected fatty acid biosynthesis. Similar effects of temperature and water availability on fatty acid composition have been reported under Mediterranean environments [1, 15], emphasizing the importance of multi-year evaluations for selecting genotypes with stable oil quality.

### Principal component analysis

Principal component analysis (PCA) was performed to investigate the relationships among agronomic traits and fatty acid composition parameters and to visualize the diversity among safflower genotypes. The eigenvalue analysis revealed that the first three principal components had eigenvalues greater than 1.0 and together explained 68.35% of the total variation. The first principal component (PC1) accounted for 38.62% of the variation, whereas PC2 and PC3 explained 17.85% and 11.88%, respectively (Supplementary Table S1 and Figure S1). Therefore, the first two principal components cumulatively explained 56.47% of the total variation and were considered sufficient to describe the major patterns of diversity among genotypes.

According to the loading matrix, oleic acid, linoleic acid, head number, number of branches, and stearic acid were the major contributors to PC1 (Supplementary Table S2). Linoleic acid (0.426) and stearic acid (0.352) had strong positive loadings, whereas oleic acid (-0.432), number of heads per plant (-0.370), number of branches (-0.357), seed yield (-0.270), and thousand seed weight (-0.261) exhibited strong negative loadings. The opposite orientation of oleic and linoleic acid vectors in the variable correlation circle (Fig. 1) clearly demonstrated the strong inverse relationship between these two fatty acids. This finding is consistent with the well-known biochemical conversion of oleic acid to linoleic acid through the FAD2 desaturase pathway and agrees with recent studies reporting that fatty acid composition represents the major source of variation in safflower germplasm [9, 14].

The second principal component (PC2) was mainly influenced by head diameter (-0.527), plant height (-0.502), and seeds per head (-0.421), indicating that this axis primarily represented variation in plant architecture and reproductive traits. In contrast, PC3 was largely associated with thousand-seed weight (0.656) and seeds per head (-0.513), reflecting variation related to seed size and seed number ( Supplementary Table S2). 

The PCA score plot revealed substantial variation among genotypes and years (Fig. 1). Several genotypes occupied distinct positions in the multivariate space, indicating considerable diversity in agronomic and quality characteristics. The cultivar Asol was clearly separated from most other genotypes and was strongly associated with the oleic acid vector in the biplot (Fig. 2), confirming its characteristic high-oleic oil profile. Similarly, Olas was positioned close to Asol and exhibited a comparable fatty acid composition pattern.

The contribution of individual traits to PC1 further supported this interpretation (Supplementary Figure S2). Oleic acid and linoleic acid were the most influential variables, each contributing approximately 18% to PC1, followed by number of heads per plant, number of branches, and stearic acid. These results indicate that both oil quality and yield-related traits played major roles in differentiating the evaluated genotypes. Similar observations were reported by Sajid et al. [18], who identified fatty acid composition and yield components as the principal discriminating variables among safflower accessions evaluated using multivariate analyses.

In contrast, several gene bank accessions, including TR 42667, TR 50118, TR 49117, and TR 51504, were located on the positive side of PC1 and were associated with higher linoleic and stearic acid contents (Fig. 2). This separation demonstrates the existence of substantial genetic variability within the evaluated Anatolian safflower germplasm and highlights the potential of these accessions for breeding programs targeting specific oil quality traits. High-oleic genotypes are generally preferred for improved oxidative stability and industrial applications, whereas high-linoleic genotypes are considered valuable for nutritional purposes because of their higher essential fatty acid content.

The PCA biplot also showed a partial separation between observations recorded in 2020 and 2021. Although the overall clustering pattern remained similar, environmental conditions appeared to influence the expression of agronomic and quality traits. Previous studies have demonstrated that temperature and moisture conditions during flowering and seed development significantly affect fatty acid biosynthesis and modify the balance between oleic and linoleic acids in safflower [8, 15, 17]. The observed year-to-year shifts in genotype positions therefore suggest a degree of environmental influence on trait expression while simultaneously confirming the presence of stable genetic differentiation among genotypes.

Overall, PCA effectively summarized the complex relationships among agronomic and quality traits and demonstrated that fatty acid composition, particularly the oleic–linoleic trade-off, together with yield-related traits, represented the major sources of variation among the evaluated safflower genotypes. The wide dispersion of genotypes in the multivariate space confirms the breeding value of Anatolian safflower germplasm and highlights its potential for the development of cultivars combining desirable agronomic performance with improved oil quality.

## Conclusion

This study demonstrated the existence of substantial genetic variation among Anatolian safflower (*Carthamus tinctorius* L.) germplasm and registered cultivars for agronomic performance, oil content, and fatty acid composition under Mediterranean winter-growing conditions. Significant differences among genotypes were observed for most yield-related and oil-quality traits, highlighting the potential of Anatolian genetic resources for safflower improvement.

Among the evaluated accessions, TR 42670 was distinguished by its superior seed and oil yield performance, whereas TR 50118 exhibited the highest oil content and TR 49117 was characterized by elevated linoleic acid concentration. In addition, the commercial cultivars Linas, Olas, Balcı, and Asol showed favorable performance for specific agronomic and oil-quality traits. These findings indicate that both local germplasm and registered cultivars possess promising characteristics that can be utilized in future breeding programs.

Principal component analysis further revealed that fatty acid composition, particularly the oleic–linoleic acid balance, together with yield-related traits, represented the major sources of variation among genotypes. The clear differentiation between high-oleic and high-linoleic materials demonstrated the presence of considerable diversity for oil quality traits within the evaluated germplasm.

Overall, the results indicate that Anatolian safflower germplasm represents a promising genetic resource for the simultaneous improvement of seed yield, oil content, and fatty acid composition. These genetic materials have considerable potential for use as parental germplasm in breeding programs aimed at developing high-yielding, superior oil-quality cultivars adapted to Mediterranean environments, where increasing climatic variability and water scarcity pose major challenges to sustainable crop production. Nevertheless, further multi-location and multi-environment evaluations are required to verify the stability, adaptability, and breeding value of the most promising genotypes.

## Supplementary Information


Supplementary Material 1.


## Data Availability

You can reach raw datas: https://docs.google.com/spreadsheets/d/1Jxwbf9ty97Q39oOQOe9ujbAp55CLY8p7/edit?usp=sharing&ouid=100147643190982780097&rtpof=true&sd=true, https://docs.google.com/spreadsheets/d/1ufUY8Be7UqBOqhAwCFKbQJxUeW_cKyub/edit?usp=sharing&ouid=100147643190982780097&rtpof=true&sd=true. Also All data supporting the findings of this study are summarized in the article in the form of ANOVA and LSD tables. If requested, the raw data is available on my computer’s hard drive and I can send it at any time.
