# Supplementary material for: Agronomic performance, oil quality, and multivariate characterization of Anatolian safflower (Carthamus tinctorius L.) germplasm under Mediterranean winter-growing conditions
Source: BMC Plant Biol. 2026 Jul 25;26:1484. doi: 10.1186/s12870-026-09499-2 (PMC13523343; doi:10.1186/s12870-026-09499-2)
Supplement: Supplementary file 1 [file 12870_2026_9499_MOESM1_ESM.docx]

Suplementary Material

Table S1. Eigenvalues, explained variance and cumulative variance of the principal components derived from agronomic and quality traits.

| **Eigenvalue** | **Variance (%)** | **Cumulative variance (%)** |
| --- | --- | --- |
| 4,248 | 38,622 | 38,622 |
| 1,964 | 17,852 | 56,474 |
| 1,307 | 11,879 | 68,353 |
| 0,971 | 8,827 | 77,180 |
| 0,873 | 7,940 | 85,120 |
| 0,560 | 5,092 | 90,212 |
| 0,385 | 3,503 | 93,715 |
| 0,285 | 2,593 | 96,308 |
| 0,264 | 2,401 | 98,709 |
| 0,142 | 1,287 | 99,996 |
| 0,000 | 0,004 | 100,00 |

Table S2. Loadings of agronomic and quality traits on the first three principal components.

| **Variable** | **PC1** | **PC2** | **PC3** |
| --- | --- | --- | --- |
| Plant_height | -0,10028 | -0,50238 | 0,290989 |
| Number_of_branches | -0,35735 | -0,07057 | -0,26538 |
| Heads | -0,37014 | -0,1252 | -0,18106 |
| Head_diameter | -0,22834 | -0,52737 | -0,10448 |
| Seeds_per_head | 0,098242 | -0,42101 | -0,51288 |
| Seed_yield | -0,26958 | -0,1187 | 0,262186 |
| TSW | -0,2607 | -0,07671 | 0,655921 |
| Linoleic | 0,425981 | -0,09312 | 0,106397 |
| Oleic | -0,43201 | 0,121546 | -0,10416 |
| Palmitic | 0,174702 | -0,40292 | 0,106282 |
| Stearic | 0,352421 | -0,25658 | 0,075622 |

Figure S1. Variable correlation circle showing the relationships among agronomic traits and fatty acid composition parameters based on the first two principal components (PC1 and PC2).

Figure S2. Relative contribution (%) of agronomic and quality traits to the first principal component (PC1).
